# Pilot randomized trial of therapeutic hypothermia with serial cranial ultrasound and 18-22 month follow-up for neonatal encephalopathy in a low resource hospital setting in uganda: study protocol

**DOI:** 10.1186/1745-6215-12-138

**Published:** 2011-06-04

**Authors:** Nicola J Robertson, Cornelia F Hagmann, Dominique Acolet, Elizabeth Allen, Natasha Nyombi, Diana Elbourne, Anthony Costello, Ian Jacobs, Margaret Nakakeeto, Frances Cowan

**Affiliations:** 1 Institute for Women's Health, 86-96 Chenies Mews, University College London, London, WC1E 6HX, UK; 2 Uganda Women's Health Initiative, University College London, London, UK; 3 Department of Pediatrics and Imaging Sciences, Imperial College Comprehensive Biomedical Research Centre, London, UK; 4 Medical Statistics Unit, London School of Hygiene and Tropical Medicine, London, UK; 5 Special Care Baby Unit, Mulago Hospital, Makerere University, Kampala, Uganda; 6 International Perinatal Care Unit, Institute of Child Health, London, UK

## Abstract

**Background:**

There is now convincing evidence that in industrialized countries therapeutic hypothermia for perinatal asphyxial encephalopathy increases survival with normal neurological function. However, the greatest burden of perinatal asphyxia falls in low and mid-resource settings where it is unclear whether therapeutic hypothermia is safe and effective.

**Aims:**

Under the UCL Uganda Women's Health Initiative, a pilot randomized controlled trial in infants with perinatal asphyxia was set up in the special care baby unit in Mulago Hospital, a large public hospital with ~20,000 births in Kampala, Uganda to determine:

(i) The feasibility of achieving consent, neurological assessment, randomization and whole body cooling to a core temperature 33-34°C using water bottles

(ii) The temperature profile of encephalopathic infants with standard care

(iii) The pattern, severity and evolution of brain tissue injury as seen on cranial ultrasound and relation with outcome

(iv) The feasibility of neurodevelopmental follow-up at 18-22 months of age

**Methods/Design:**

Ethical approval was obtained from Makerere Unive**r**sity and Mulago Hospital. All infants were in-born. Parental consent for entry into the trial was obtained. Thirty-six infants were randomized either to standard care plus cooling (target rectal temperature of 33-34°C for 72 hrs, started within 3 h of birth) or standard care alone. All other aspects of management were the same. Cooling was performed using water bottles filled with tepid tap water (25°C). Rectal, axillary, ambient and surface water bottle temperatures were monitored continuously for the first 80 h. Encephalopathy scoring was performed on days 1-4, a structured, scorable neurological examination and head circumference were performed on days 7 and 17. Cranial ultrasound was performed on days 1, 3 and 7 and scored. Griffiths developmental quotient, head circumference, neurological examination and assessment of gross motor function were obtained at 18-22 months.

**Discussion:**

We will highlight differences in neonatal care and infrastructure that need to be taken into account when considering a large safety and efficacy RCT of therapeutic hypothermia in low and mid resource settings in the future.

**Trial registration:**

Current controlled trials ISRCTN92213707

## Background

In the UK and other developed world settings, moderate-severe encephalopathy following perinatal asphyxia occurs in 1-2/1000 term births [1]. There are major consequences for families and for society; approximately 25% of affected infants will die in the neonatal unit and 40% will develop cerebral palsy. Others will have long-term neurodevelopmental deficits that result in a difficult life and lost potential [2]. Within the last decade, therapeutic hypothermia for infants with perinatal asphyxial encephalopathy has been studied in several randomised controlled trials (RCTs) in industrialized countries [3-6]. Meta-analyses of these trials show that therapeutic hypothermia increases survival with normal neurological function (pooled risk ratio of 1.53) with a number needed to treat of 8 (95% confidence interval (CI) 5 - 17) and in survivors reduces the rates of severe disability and cerebral palsy [7,8]. Therapeutic hypothermia under intensive care settings appears safe [7]. Therapeutic hypothermia is now widely offered to moderately or severely asphyxiated infants in high-income countries [9]. In 2010 in the UK the National Institute for Health and Clinical Excellence (NICE) endorsed *Therapeutic Hypothermia with Intracorporeal Temperature Monitoring for Hypoxic Perinatal Brain Injury *(see website: http://www.nice.org.uk/nicemedia/live/11315/48809/48809.pdf)

The global burden of disease estimates indicate that perinatal asphyxia is a very significant problem in low and mid-resource settings [10]; for example in sub-Saharan Africa, neonatal encephalopathy related to perinatal asphyxia is ~10-20 times more common than in the developed world [11]. Globally, perinatal asphyxia is responsible for 42 million disability life adjusted years, double that due to diabetes and three quarters of that due to HIV/AIDS [10]. Almost one quarter of the world's 4 million annual neonatal deaths are caused by perinatal asphyxia [12] and 99% of these deaths occur in low and mid-resource settings. These intrapartum-related deaths account for as many deaths as does malaria but they are not addressed in any global health policy.

A safe and effective therapy for neonatal encephalopathy appropriate for low resource settings is likely to benefit millions of infants and families. There are, however, several compelling reasons why the efficacy and safety data on therapeutic hypothermia from high-income countries cannot be simply extrapolated to mid and low resource settings. For example **(i) **brain injury may be already established due to multiple antenatal insults (e.g. maternal malnutrition and other co-morbidities), **(ii) **prolonged obstructed labour, long delays in carrying out emergency caesarean sections, and lack of effective resuscitation and networks for neonatal transport may all reduce or nullify the therapeutic window for hypothermia [13]. **(iii) **the incidence and profile of perinatal infections in this population is different. Cooling in the presence of infection might even be deleterious as hypothermia may impair innate immune function, including neutrophil migration and function [14], although it is reassuring that the neonatal cooling trials from industrialized countries did not show a higher incidence of sepsis in cooled infants. It is important to note that hypothermia during sepsis in adult patients has been associated with increased mortality and higher circulating levels of TNF-a AND IL-6 [15]. One explanation for the higher morbidity and mortality associated with hypothermia in some clinical settings may be the prolongation of NF-KB activation and altered cytokine gene expression known to occur with moderate hypothermia [16]. Furthermore, there is convincing experimental [17,18] and epidemiological evidence [19,20] suggesting that a 'dual hit' of infection and ischaemia results in disproportionately more severe brain injury and increases in the risk of cerebral palsy. It is likely that this 'dual hit' is one of the factors responsible for the poorer neurological outcome reported from low and mid-resource settings [21] and it is not known if therapeutic hypothermia would be neuroprotective in such situations. **(iv) **cooling may be unsafe in the presence of meconium aspiration and pulmonary hypertension where facilities for advanced multi-organ support may not be available; **(v) **the benefits of cooling may only be achieved in units with optimal neurological monitoring which may not be possible in low income settings. **(vi) **cooling equipment used in high income countries is expensive, requires maintenance support and has ongoing costs. There is therefore an important need for validation of 'low tech', safe and economical cooling methods.

Uganda was chosen as an optimal site for the pilot study because of the long tradition of research at Mulago Hospital and Makerere University. In 2006 the UCL Uganda Women's Health Initiative was set up between UCL and Mulago Hospital, Makerere University and Hospice Africa. Ten projects related to women's health were set up under the Uganda Women's Health initiative. One of the neonatal projects initially focused on training midwives in neonatal resuscitation, however MN was very keen to help the large number of infants with perinatal asphyxia admitted to the special care baby unit at Mulago each week. We set up a pilot randomized trial of therapeutic hypothermia in Mulago Hospital, Uganda to determine the feasibility of a larger RCT in this setting.

The aims of the pilot study were to determine:

(i) The feasibility of achieving consent, neurological assessment, randomisation and whole body cooling to a core temperature 33-34°C using a 'low tech' cooling method (water bottles) (already briefly reported [22])

(ii) The temperature profiles of encephalopathic infants randomized to standard care or standard care plus therapeutic hypothermia (already briefly reported [22])

(iii) The pattern, severity and evolution of brain tissue injury as seen on cranial ultrasound and relation with outcome

(iv) The feasibility of neurodevelopmental follow-up at 18-22 months of age

## Methods/design

### Study Design

Ethical Approval was obtained from the Ethics Committees of the Medical School, Makerere University and Mulago Hospital.

#### Training and teaching Ugandan staff

**Figure 1:**
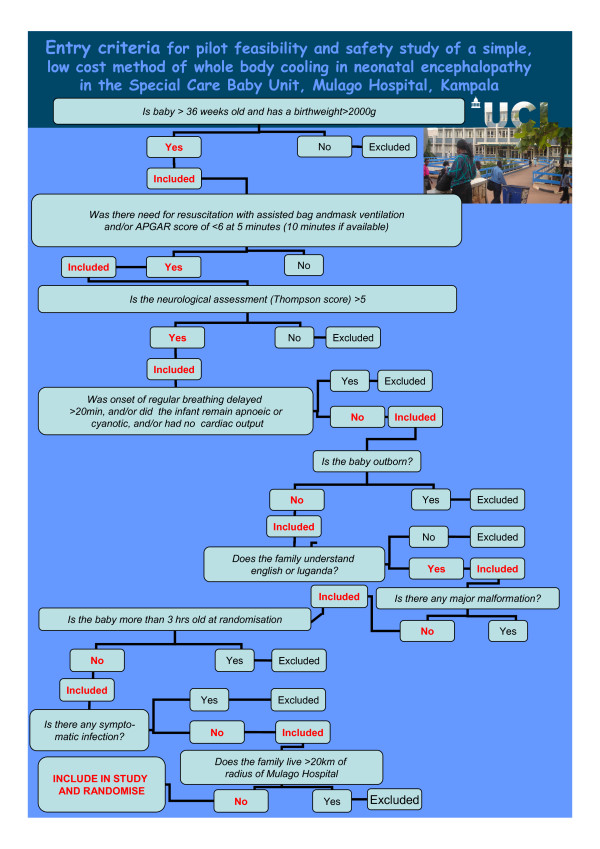
**Entry criteria for pilot feasibility and safety study of a simple, low cost method of whole body cooling in a neonatal encephalopathy in the Special Care Baby Unit, Mulago Hospital, Kampala**.

**Figure 2:**
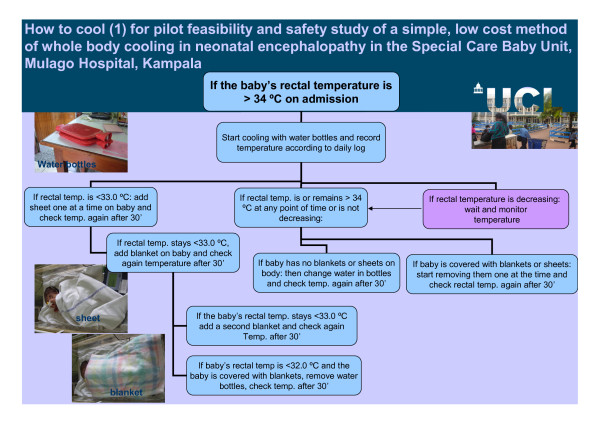
**How to cool (1) for pilot feasibility and safety study of a simple low cost method of whole body cooling in a neonatal encephalopathy in the Special Care Baby Unit, Mulago Hospital, Kampala**.

**Figure 3:**
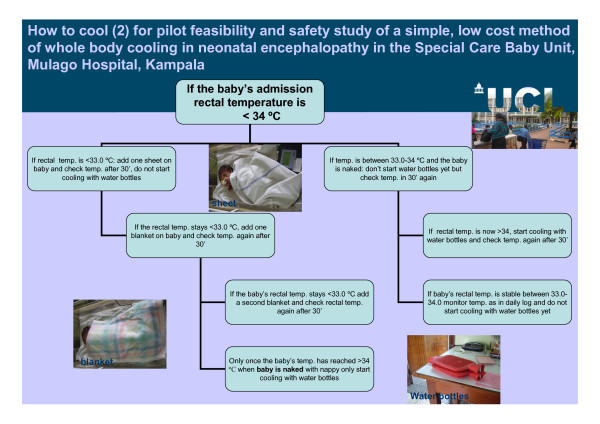
**How to cool (2) for pilot feasibility and safety study of a simple low cost method of whole body cooling in a neonatal encephalopathy in the Special Care Baby Unit, Mulago Hospital, Kampala**.

**Figure 4:**
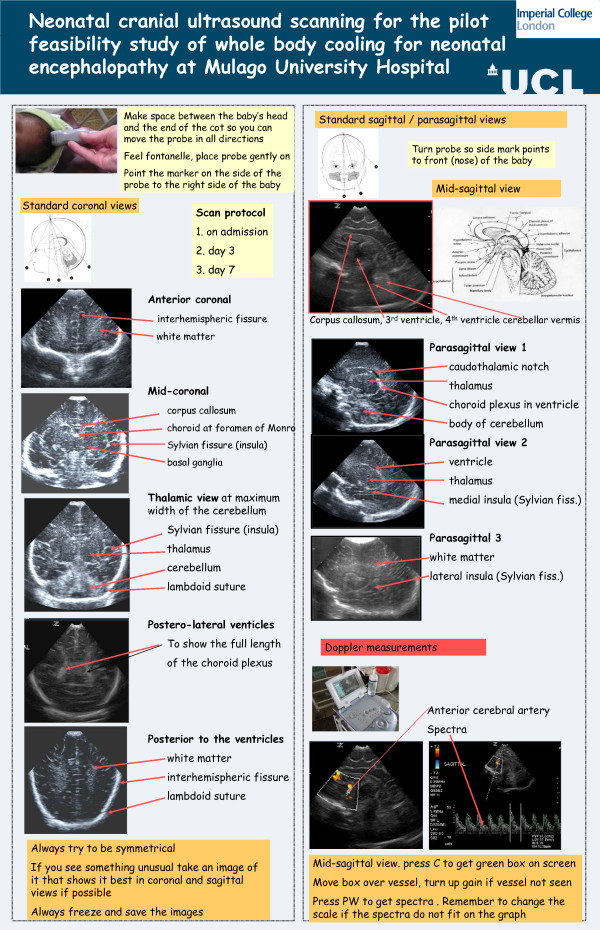
**Neonatal cranial ultrasound scanning for the pilot feasibility and safety study of a simple, low cost method of whole body cooling for neonatal encephalopathy at Mulago Hospital**, Kampala

NR, DA, CH and FC spent 2 weeks on the neonatal unit at Mualgo Hospital training the neonatal unit doctors and nurses about the background to the study, use of equipment, data recording and examination of patients. Six nurses were chosen as study nurses; their responsibilities included recording demographic and physiological data, changing water bottles and swaddling with blankets as required to maintain the rectal temperature within target range, general nursing care and support for the families. The nurses also recruited babies and obtained informed consent There were clear protocols and algorithms for recruitment of patients (Figure 1), cooling to the target temperature (Figures 2 and Figure 3), scoring the Thompson encephalopathy score (Table 1) [23], performing the structured neurological examination [24] and guidelines for the cranial ultrasound protocol for essential views (Figure 4). Ten other helper neonatal nurses facilitated the study by helping with the care of the other patients on the special care baby unit. Three senior doctors (MN, NN and JM) were responsible for taking informed consent from the family and for performing the Thompson score for entry eligibility and on days 1-4, the standardized and scorable neurological examination on days 7 and 17 and the cranial ultrasounds on days 1,3 and 7. CH visited for 3 weeks mid-way through the study to help with continuing training and quality control.

**Table 1:** Neonatal encephalopathy score [23].

Sign	0	1	2	3
Tone	Normal	Hyper	Hypo	Flaccid

Level of consciousness	Normal	Hyperalert, stare	Lethargic	Comatose

Fits	Normal	Infrequent <3/day	Frequent >2/day	

Posture	Normal	Fisting, cycling	Strong distal flexion	Decerebrate

Moro	Normal	Partial	Absent	

Grasp	Normal	Poor	Absent	

Suck	Normal	Poor	Absent/ bites	

Respiration	Normal	Hyperventilation	Brief apnoea	Apnoeic

Fontanelle	Normal	Full, not tense	Tense	

The score is derived from the clinical assessment of 9 signs. Each sign is scored from 0 to 3 and the score for each day is totalled. The higher the score the more severely affected the infant. The maximum possible score is 22.

#### Study size and overall plan

This study was intended to be a feasibility study to assess the practical aspects of setting up a RCT of therapeutic hypothermia using 'low tech' cooling methods in a low resource hospital setting. The study was not intended to assess efficacy of hypothermia as a neuroprotective treatment and was not powered for this, although it was envisaged that the pilot study might provide information about sample size for a future larger RCT. The aim was to recruit ~40 infants to this study in line with previous pilot studies of cooling performed in industrialized countries [25-27].

#### Feasibility

This high incidence of perinatal asphyxial encephalopathy and feasibility of recruiting ~40 infants within 3 months was shown by a previous admissions audit at Mulago Hospital in 2006. During this period there were 13,500 deliveries, 1,740 admissions to the neonatal unit and 239 infants with moderate to severe neonatal encephalopathy (giving an incidence of moderate to severe neonatal encephalopathy of 18/1000 live births).

#### Inclusion criteria (Figure 1)

Study infants were near term or full term infants with:

1. Need for resuscitation with assisted bag and mask ventilation after birth and/or an Apgar score of <6 at 5 minutes (10 minutes if available)

2. Abnormal Thompson neonatal encephalopathy assessment score of >5 between 30 min and 3 h of age [20].

#### Exclusion criteria (Figure 1)

1. <36 weeks gestation or birthweight <2000 g

2. Imminent death anticipated at the time of assessment for eligibility

3. >3 h of age at study entry

4. Major malformations

5. Symptomatic infection

6. Outborn

7. Families who live >20 km away from Mulago Hospital

8. Parents who did not speak or understand either English or Luganda or any parent who does not understand the study.

#### Consent

Informed consent was obtained using an information sheet available in either English or Lugandan. The study doctor met with the parents in the intervention period to ensure they understand the study procedures and continue to consent to participation in the study [28].

#### Randomization

Randomization was by sealed envelopes in eligible infants up to a total of 4 study infants at a time. The maximum number of infants we were able to study at any one time was 4 due to the limited number of available data loggers and the need to keep the workload to an acceptable level.

##### Standard care group

Infants receiving standard care were cared for in cots according to usual hospital clinical practice. Standard care at Mulago Hospital Neonatal Unit included:

Oxygen saturation monitoring: depending on the availability of the only pulse oximeter in the neonatal unit. Oxygen saturations were measured on each infant 4-12 hrly by moving the oxygen saturation monitor from one infant to another. If an infant looked cyanotic, supplementary nasal cannulae oxygen was given if at least 2 recordings of a saturation <85% had been recorded.

Blood pressure: it was not possible to measure mean arterial blood pressure.

Temperature monitoring: Rectal, axillary, ambient and water bottle surface temperatures were monitored continuously and stored using a 4-channel data logger (Squirrel SQ 2020, Keison Products, Chelmsford, UK). No intervention other than adding or removing cotton sheets was used to adjust the rectal temperature to normothermia.

Administration of antibiotics: The current neonatal unit guidelines at Mulago Hospital include administration of Ampicillin and Gentamicin to all sick infants.

Blood glucose and fluid administration: An IV cannula was sited and 10% dextrose was given intravenously as hourly boluses to make up 60 ml/kg/day according to the current neonatal unit guidelines. Blood glucose was measured every 6 h using glucose test strips (Optimum Medisense H Blood Glucose Test strips and calibrator, Abbott Diabetes Care Ltd, Oxon, UK).

Sedation: If infants appeared agitated or unsettled, phenobarbitone 20 mg/kg IV was given.

Treatment of clinical seizures: administration of an intravenous bolus of 10% dextrose. If seizures persisted then 20 mg/kg phenobarbitone was given intravenously and repeated if necessary.

##### Standard care plus cooling (Figures 2 and 3)

Infants randomized to standard care plus cooling were cared for in exactly the same way as standard care apart from the temperature control. Infants were nursed in a cot naked or lightly covered with a cotton sheet. Active cooling was by placing a mattress under the infant - the mattress comprised three commercially available water bottles filled with tepid tap water (25°C) and covered in a cotton sheet. The tap water temperature was fairly stable throughout the 24 hours and generally around 22-25°C. The sheet-covered water bottles were in contact with as much of the skin surface area of the infant as possible. The target rectal temperature was 33-34°C. The rectal, axillary, ambient and surface water bottle temperatures were monitored continuously and stored using the data logger. Our pre-clinical work suggested that the bottle water would need to be changed every 8-12 h [29]. If warming was required, the infant was dressed or covered with blankets and sheets as required. Cooling was concluded 72 hours after randomization or earlier if clinical circumstances dictated. The rectal temperature was then be allowed to rise by no more than 0.5°C per hour to 37°C, by slowly reducing the contact of the baby with the water bottles. This was achieved by progressively removing water bottles from beneath the baby and swaddling the baby. All other aspects of clinical care were the same in both groups.

#### Early Neurological assessment

The severity of the infant's encephalopathy was assessed and scored daily for the first 4 days using the Thompson score (Table 1) [23]. A standardized neurological examination was performed on days 7 and 17 and a neurological optimality score calculated (the score was adapted from the published optimality score norms - total maximum score 34) [24,30]. This examination was simple to teach and required that the predominant patterns of posture/tone/reflex activity/movements and behavior were circled and could be scored later [24]. The head circumference was measured at birth and on day 7 and 17 [31].

#### Cranial ultrasound

A hand portable ultrasound machine (Z.one Ultra Convertible Ultrasound System, 3.0 Release, Zonare Medical Systems Inc. USA) was used in this study. Infants were scanned within the first 24 h of admission (to determine possible antenatal brain injury) and at 3 and 7 days. Practical training sessions on the use of the machine and the optimal views to be taken were undertaken with the Uganda doctors prior to the start of the study (Figure 4).

The ultrasound scans were analyzed according to a graded scoring system which assessed (i) basal ganglia and thalami (scale 0-3) and (ii) white matter; (scale of 0-4) with 0 being normal and 4 being most abnormal and (iii) cortex (scale 0-3) (Table 2). Any other abnormality was noted e.g. cortical abnormality, sub-ependymal or choroidal cysts, lenticulostriate vasculopathy or other calcification, ventricular size evidence of atrophy. The scores for each brain area on the latest cranial ultrasound scan were added to give a total cranial ultrasound score.

**Table 2:** Cranial ultrasound scoring system for basal ganglia, white matter and cortex

Score	Basal ganglia and thalamus	White matter	Cortex
**0**	Normal	Normal	Normal

**1**	Internal capsule seen	Mildly echogenic patchy parietal echogenicity	Focal sulcal highlighting

**2**	Mild - swollen and/or small focal unilateral abnormality	More diffuse parieto-temporal echogencity	Diffuse sulcal highlighting

**3**	Clearly demarcated focal bilateral echodensity	Diffuse white matter echogenicity	Cortical echogenicity and thickening

**4**	-	Cystic changes	

To determine the incidence of abnormalities seen on cranial ultrasound in a population of low-risk African babies, ~100 newborn infants on the postnatal ward underwent a scan using the standard 11 views as soon as possible after birth [32].

#### Reporting procedures for adverse events

Any adverse events (especially if serious and/or unexpected) occurring during the study whether or not attributed to the study intervention were reported in a timely manner following local regulatory procedures.

#### Neurodevelopmental follow-up

Where possible a health visitor took the family home so that the house location was known for subsequent tracing if no address available. Mobile phone numbers and the village where the family lived were recorded. Contact was kept as far as possible with the family when they returned to the hospital for the childhood immunizations at the following ages:

**6 weeks, 10 weeks, 14 weeks: **DPT OPV vaccines

**6 months, 9 months**: measles vaccine

**12 months: **measles vaccine.

No neurodevelopmental assessment was performed at these ages, but the family were seen by MN and health concerns could be discussed and advice given.

Neurodevelopmental assessment was performed by FC and CH at 18-22 months using the Griffiths developmental quotient [33], neurological examination and assessment of gross motor function (GMFS). Head circumference was measured [31]. Local health workers with experience in neurological assessment of newborn infants helped with communication in the local language. The Griffiths developmental quotient was chosen for this study as it has been standardized [34] and validated in a multiracial South African setting, and used for assessment of outcome [23]. The infant neurological exam can be scored as a record of motor abilities [35,36]; the gross motor function classification gives functional motor outcome [37,38] and is a standardized scale recognized world-wide for this purpose.

Intact survival was defined as all of DQ >84, no neuromotor impairment (neurological exam score ≥73 and GMFCS ≤I), normal vision and hearing on clinical examination.

### Statistical analysis

Demographic factors and clinical characteristics were summarized with counts (percentages) for categorical variables, mean (standard deviation [SD]) for normally distributed continuous variables or median (interquartile or entire range) for other continuous variables. The temperature data from the standard care and standard care plus therapeutic hypothermia groups were compared using t-tests. The Thompson scores, the neurological exam scores at 17 days and the cranial ultrasound scores were compared with neurodevelopmental outcome at 18-22 months using appropriate regression analysis that will allow for repeated measures.

## Results

Partial data from this trial have been reported briefly elsewhere [22]. There were three main points reported in this correspondence: (i) Therapeutic hypothermia with whole body cooling using a low tech, low cost cooling device (water bottle filled with tepid tap water form the neonatal unit) is feasible in a low resource setting. (ii) The rectal temperature of standard care infants was hypothermic for a mean of 15.6 (standard deviation 14.6)h after birth in this setting where only swaddling and gloves filled with hot water are used to keep babies warm. Such passive cooling in term babies with perinatal asphyxia was first described some 50 years ago; babies with 'asphyxia' (defined as a failure to establish respiration within 3 min of birth) were 2°C cooler than non-asphyxiated infants for up to 16 h after birth [39]. This phenomenon may be due to impairment in non-shivering thermogenesis in asphyxiated infants, which is controlled centrally by the hypothalamus. Larger trial sizes might be required in this setting due to dilution of the hypothermic effect in the cooled group by the standard care group. Such "passive" cooling in the standard care group in this setting where the only method of re-warming was swaddling differs to the developed world trials where at least one third of the standard care infants had an elevated core temperature during the first 3 days [4,40]; (iii) Higher mortality was seen in the cooled vs the standard care group (risk ratio: 5.0 (95% confidence interval (CI) 0.7-37)[8], absolute difference: 0.267 (95% CI 0.029, 0.505). More infants with severe neonatal encephalopathy were randomized to the cooled group, which could explain the excess deaths; nevertheless the possibility of infection related deaths can't be excluded, as there were no facilities for infection screening at the time. Importantly, although the study was not powered to look at safety or efficacy, these findings underline the importance of exerting caution in the application of therapeutic hypothermia in low and mid resource settings where the patient risk factors and environment are very different.

## Discussion

### Cranial ultrasound, neurological assessment and neurodevelopmental outcome

Cranial ultrasound has been used extensively in neonatal practice in the developed world, since it is a convenient technique to visualize the neonatal brain through the anterior fontanelle serially without moving or disturbing the patient. Cranial ultrasound is helpful to exclude structural abnormality suggesting metabolic and other diagnoses, detect calcification and cysts suggestive of viral infection and detect atrophy suggestive of long standing damage. It will also identify cerebral haemorrhage. Sequential observation of the evolution of injury following a hypoxic ischaemic insult at birth is helpful for defining the pattern and severity of lesions [41], although MRI is the imaging modality of choice to accurately define the pattern and severity of injury and prognosticate outcome [42,43].

Access to MRI is not possible in most low resource hospital settings. In this proposed study, serial cranial ultrasound studies will provide some evidence about the incidence of established injury and the evolving pattern and severity of injury in relation to the outcome. Cranial ultrasound is able to provide some very useful information on the pattern and severity of brain injury following perinatal asphyxia - for example a clear and persistent abnormality of the thalami and basal ganglia on cranial ultrasound correlates with MRI findings, which indicate a poor prognosis [44]. Other patterns of brain injury such as the watershed injury are, however, more difficult to detect on cranial ultrasound.

This is the first study of therapeutic hypothermia in a low resource setting that will include detailed cranial ultrasound imaging, have a comparative control or typical population [32] and a neonatal neurological assessment in the 3^rd ^week after birth and at 18-22 months.

### Cooling in low and mid resource settings

In early 2000 when therapeutic hypothermia was being considered as a therapy in industrialized countries, initial studies consisted of pilot feasibility studies in ~40 patients [25-27]. These pilot studies were necessary before moving onto large RCTs in industrialized countries [3,4,6]. We suggest that the same progression is needed in both low and mid-resource settings.

This small pilot study suggests that in settings such as Uganda, future studies need to focus on identifying risk factors for NE (particularly the role of perinatal infection in the aetiology and outcome of NE); we are currently engaged in such case controlled studies at Mulago Hospital. In such low resource settings with high rates of neonatal mortality it may be inappropriate to implement cooling without acceptable standards of antenatal care, delivery, resuscitation, respiratory management and infection control; effective dissemination of knowledge of best clinical practice may bring about greater reduction in asphyxia related mortality and morbidity initially [10]. Once these practices are introduced and maintained, clinical trials may be the next stage for a more sustained long-term improvement in neonatal outcome.

In mid resource settings and especially in those with a good infrastructure for resuscitation, neonatal care and follow-up, such as in parts of Asia and South America, a measured and careful approach is needed to ensure that the highest quality trials of safety and efficacy of therapeutic hypothermia are performed. It is possible that unless a trial is performed that is relevant to mid resource settings, there will be a creeping, uncertain introduction of hypothermia, with constant worries regarding residual safety concerns [45]. Furthermore, given the sheer magnitude of the problem of perinatal asphyxia and NE worldwide [12], the establishment of an effective and safe therapy would have a high impact on medical practice throughout the world, save lives and reduce the burden on our societies. In 2009, we performed a cooling feasibility study in India [46] and hope that a large Indian RCT of therapeutic hypothermia for neonatal encephalopathy can start in 2012.

## Competing interests

The authors declare that they have no competing interests.

## Authors' contributions

IJ, AC and NR had the idea to do this study. NR planned the study, wrote the protocol, set up the study in Mulago Hospital, led the training of the nurses and doctors and drafted the manuscript. CH made the training materials and posters, helped with the analysis of the ultrasound scans, helped with the teaching and drafting of the manuscript. FC and DA taught the neurological examination and ultrasound technique to the Ugandan doctors. DE and EA provided advice on the study design and EA did the statistical analysis for the early results. IJ set up the Ugandan Women's Institute and IJ and AC facilitated this study. MN and NN facilitated the trial at Mulago Hospital, collected consent from parents and performed neurological examinations and cUS scans. All authors read and approved the final version of the manuscript.
